# Microglia exosomal miRNA-137 attenuates ischemic brain injury through targeting Notch1

**DOI:** 10.18632/aging.202373

**Published:** 2021-01-10

**Authors:** Dianquan Zhang, Guoliang Cai, Kai Liu, Zhe Zhuang, Kunping Jia, Siying Pei, Xiuzhen Wang, Hong Wang, Shengnan Xu, Cheng Cui, Manchao Sun, Sihui Guo, Wenli Song, Guofeng Cai

**Affiliations:** 1 Department of Rehabilitation Medicine, Shenzhen Longhua District Central Hospital, Shenzhen, China; 2 Postdoctoral Research Workstation of Harbin Sport University, Harbin 150008, China; 3 Harbin Sport University, Harbin 150008, China; 4 Hanan Branch of Second Affiliated Hospital of Heilongjiang University of Traditional Chinese Medicine, Harbin 150001, China; 5 Second Affiliated Hospital of Heilongjiang University of Traditional Chinese Medicine, Harbin 150001, China; 6 Heilongjiang University of Traditional Chinese Medicine, Harbin, China; 7 Postdoctoral Research Station of Heilongjiang Academy of Traditional Chinese Medicine, Harbin, China

**Keywords:** microglia, ischemic injury, exosome, miRNA-137, Notch1

## Abstract

Microglia are the resident immune cells in the central nervous system and play an essential role in brain homeostasis and neuroprotection in brain diseases. Exosomes are crucial in intercellular communication by transporting bioactive miRNAs. Thus, this study aimed to investigate the function of microglial exosome in the presence of ischemic injury and related mechanism. Oxygen-glucose deprivation (OGD)-treated neurons and transient middle cerebral artery occlusion (TMCAO)-treated mice were applied in this study. Western blotting, RT-PCR, RNA-seq, luciferase reporter assay, transmission electron microscope, nanoparticle tracking analysis, immunohistochemistry, TUNEL and LDH assays, and behavioral assay were applied in mechanistic and functional studies. The results demonstrated that exosomes derived from microglia in M2 phenotype (BV2-Exo) were internalized by neurons and attenuated neuronal apoptosis in response to ischemic injury *in vitro* and *in vivo*. BV2-Exo also decreased infarct volume and behavioral deficits in ischemic mice. Exosomal miRNA-137 was upregulated in BV2-Exo and participated in the partial neuroprotective effect of BV2-Exo. Furthermore, Notch1 was a directly targeting gene of exosomal miRNA-137. In conclusion, these results suggest that BV2-Exo alleviates ischemia-reperfusion brain injury through transporting exosomal miRNA-137. This study provides novel insight into microglial exosomes-based therapies for the treatment of ischemic brain injury.

## INTRODUCTION

Microglia are the resident immune cells in the central nervous system (CNS) and play an essential role in brain homeostasis and host defense against pathogens [1]. Over the past decades, microglia have received increasing scientific interest due to their essential roles in CNS health and diseases [2, 3]. In the healthy brain, the diverse functions of microglia have been demonstrated in many previous studies, from shaping learning-related plasticity to guiding the development of neuronal circuits [2, 3]. Meanwhile, growing evidence shows that microglia are involved in many CNS diseases, for example, traumatic brain injury such as spinal cord lesions, neurogenerative disorders such as Parkinson’s diseases, and psychiatric diseases such as schizophrenia [4]. Therefore, understanding the physiological roles of microglia is critical to apply their functions in the therapies for brain diseases.

Ischemia-reperfusion injury (IRI) occurring in the brain results in irreversible loss of neurons and traumatic tissue damage in the IRI-targeted and surrounding areas [5]. Following IRI, a group of detrimental biological factors significantly emerge in the CNS, including excitatory neurotransmitters, excess ion influx, generation of free radicals, as well as inflammatory factors [6–8]. Inflammatory responses in the brain are tightly involved in the activation of microglia. Activated microglia, named M1 phenotype, secrete a mass of pro-inflammatory cytokines that exacerbating neuronal damage [6, 7]. However, other studies have reported that activated microglia, named M2 phenotype, can exert neuroprotective roles through enhancing neural proliferation and differentiation, leading to resolution of inflammation and tissue repair [7–9]. Kigerl et al. reported that microglia in M2 state promote CNS repair and attenuate secondary inflammatory-associated injury [10]. Essentially, studying the mechanism underlying the neuroprotective effect of microglia would be beneficial to develop effective treatments for IRI injury.

Exosomes, 40-100 nm in diameter, are small extracellular vesicles generated from various types of cells, including neurons, cancer cells, epithelial cells, and chondrocytes [11]. Previous studies have suggested that various bioactive components, such as lipids, proteins, mRNAs, microRNAs (miRNAs), and other non-coding RNAs (ncRNAs), have been identified in the lumen of exosomes [12]. Furthermore, these biological components can be taken up by neighboring or distant cells, then regulating the physiology of recipient cells [13]. Given such characteristics, exosomes play crucial roles in intercellular communication [13]. Regarding microglial exosomes, a study conducted by Huang et al. reported that the traumatic brain injury (TBI)-induced upregulation of exosomal miRNA-124-3p derived from microglia exerts an inhibitory effect on neuronal inflammation and promotes the neurogenesis [14]. In another study reported by Ge et al., the elevation of microglial exosomal miRNA-124-3p improves cognitive ability and alleviates neurodegeneration resulted from repetitive mild traumatic brain injury (rmTBI) [15]. Furthermore, microglial exosomes attenuate photoreceptor injury and promote angiogenesis in the animal model of retinopathy of prematurity [16]. As such, microglial exosomes have great potential for developing therapeutic strategies for brain diseases.

As mentioned above, miRNAs can be transported by exosomes between cells and play essential roles in the regulation of cellular biology [17]. MiRNAs, a class of short non-coding RNAs, can modulate gene expression through targeting 3’UTR of mRNA or promoting the degradation of mRNA [18]. It has been well-studied that miRNAs participate in various physiological and pathological processes, such as cell proliferation, autophagy, apoptosis, differentiation, tumor cell migration, and cell metabolism [18–20]. Meanwhile, the roles of exosomal miRNAs also have been reported in many diseases. For example, Vinas et al. demonstrated that exosomal miRNA-486-5p derived from human endothelial colony forming cell inhibits apoptosis of endothelial cells and attenuates ischemic injury in the kidney [21]. Also, exosomes derived from cardiac progenitor cells suppress cardiomyocyte apoptosis through exosomal miRNA-21 under oxidative stress [22]. Exosomal miRNA-25 originated from bone marrow mesenchymal stem cells can decrease malondialdehyde content and increase superoxide dismutase activity, thus protecting spinal cords in response to transient ischemia [23].

Collectively, given these previous findings, we hypothesize that microglial exosomal miRNAs might play neuroprotective effects in the mouse brain under ischemic injury. Thus, this study aimed to address our hypothesis and related mechanisms.

## RESULTS

### BV2-CM attenuated OGD-induced neuronal death

**Figure 1:**
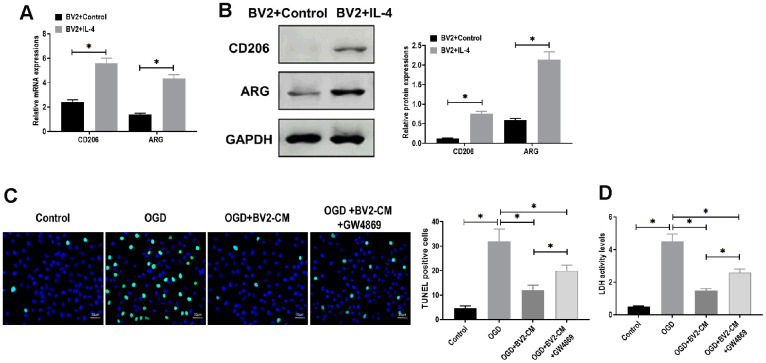
**M2-phenotype microglia-conditioned medium attenuated oxygen-glucose deprivation (OGD)-induced neuronal death**. (**A**) mRNA expressions of CD206 and ARG in BV2 cells treated with or without IL-4 treatment. (**B**) Protein expressions of CD206 and ARG in BV2 cells treated with or without IL-4 treatment. (**C**) TUNEL assay for detecting apoptosis in neurons in 1) control, 2) OGD, 3) OGD plus M2-phenotype microglia-conditioned medium (OGD+BV2-CM), and 4) OGD plus M2-phenotype microglia-conditioned medium plus exosome secretion inhibitor GW4869 (OGD+BV2-CM+GW4869). Scale bar = 20 μm. (**D**) Lactate dehydrogenase (LDH) assay for detecting LDH activity in neurons in 1) control, 2) OGD, 3) OGD+BV2-CM, and 4) OGD+BV2-CM+GW4869. Data are presented as mean±SD. *, *p*<0.05. At least three replicates were available for statistical analysis in each treatment.

Microglia in M2 phenotype play a crucial neuroprotective role in several brain diseases, such as cerebral ischemia [24] and Parkinson's disease [25]. To detmine the mechanism underlying the neuroprotective effect of microglia, BV2 cells were treated with IL-4 (20 ng/mL) to polarized to M2 phenotype [7, 26]. The results showed that both mRNA and protein expressions of CD206 and ARG, M2 microglia markers [27], were upregulated, compared with control BV2 cells (Figure 1A, 1B). In addition, we found that M2 BV2 cells-conditioned medium (BV2-CM) decreased apoptosis in neurons in the presence of OGD treatment (Figure 1C). In LDH assay, BV2-CM decreased LDH activity, indicating less cellular injury was detected in neurons after OGD treatment (Figure 1D). Intriguingly, we also observed that the addition of GW4869, an inhibitor of exosome secretion [28], partially reversed the effect of BV2-CM on OGD-treated neurons (Figure 1C, 1D). These results together suggested that BV2-derived exosomes may participate in a neuroprotective mechanism in response to OGD.

### BV2-Exo identification

**Figure 2:**
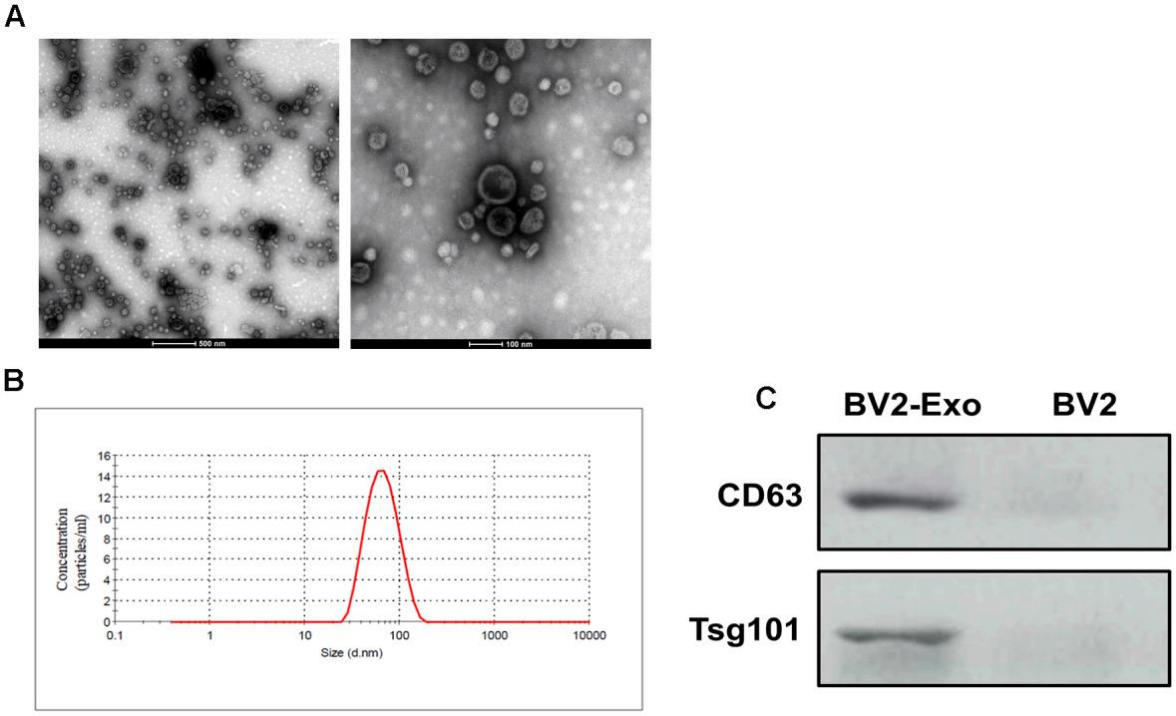
**Identification of M2-phenotype microglia-derived exosomes (BV2-Exo).** (**A**) Morphology of BV2-Exo, as determined by transmission electron microscopy. Scale bar = 500 nm (left panel) and 100 nm (right panel). (**B**) Size distribution of exosomes, as determined by nanoparticle tracking analysis. (**C**) Expression of exosome markers CD63 and Tsg101 in BV2-Exo and BV2 cells. Data are presented as mean±SD. *, *p*<0.05. At least three replicates were available for statistical analysis in each treatment.

Next, we isolated exosomes from BV2 cells in M2 phenotype through the ultracentrifugation procedure. By TEM, the morphology of exosomes displayed classic spherical shape (Figure 2A), and nanoparticle tracking analysis revealed that the size of exosomes was in the range of 40-130 nm (Figure 2B). Also, Western blotting assay suggested that the exosome markers CD63 and Tsg101 were positively expressed in the exosomes, compared with those of BV2 cells (Figure 2C). Collectively, these results indicated that we successfully isolated exosomes from BV2 cells in M2 phenotype.

### BV2-Exo attenuated neuronal apoptosis induced by OGD

**Figure 3:**
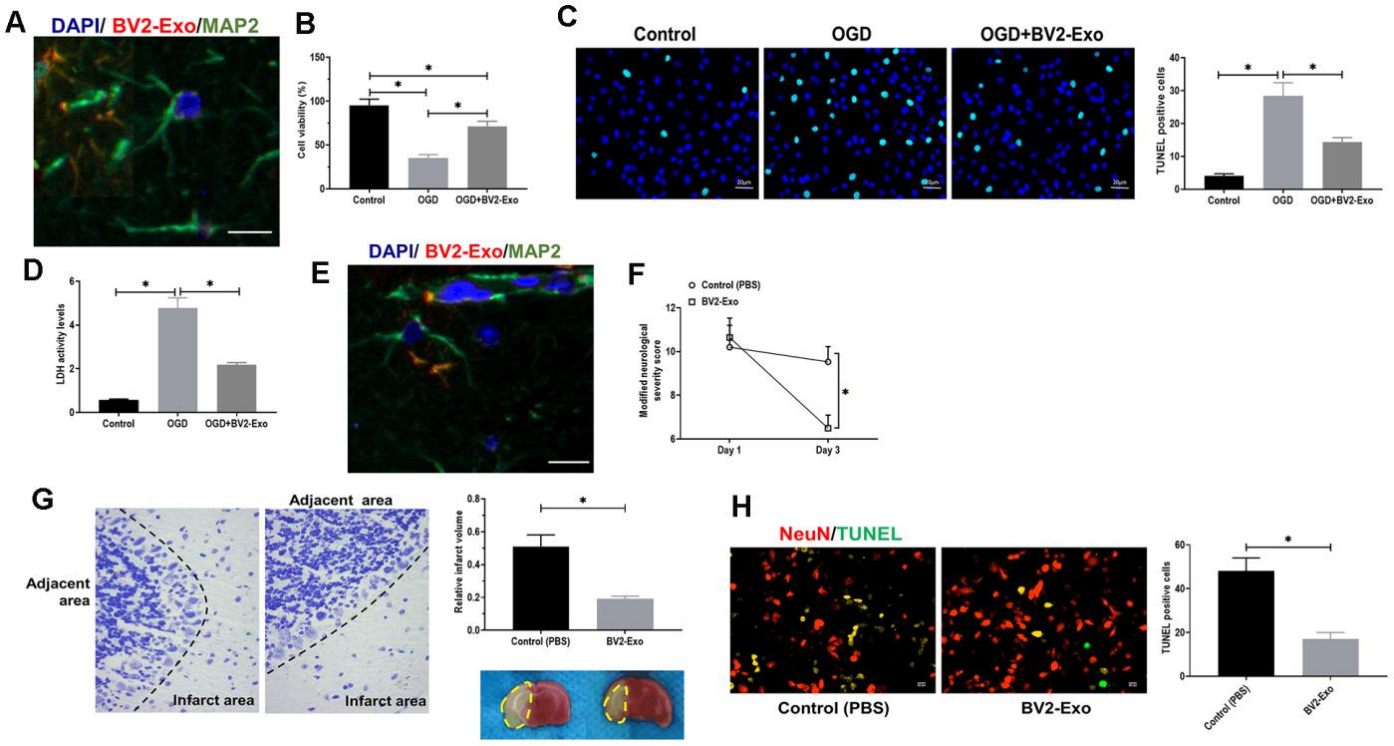
**M2-phenotype microglia-derived exosomes (BV2-Exo) attenuated neuronal apoptosis induced by ischemic injury.** (**A**) BV2-Exo was internalized by neurons *in vitro*, as imaged by confocal microscopy. Red color indicated exosomes (PKH26), blue color indicated nuclei (DAPI), and green color indicated neurons (MAP2). Scale bar = 25 μm. (**B**) Cell viability of neurons treated with OGD or OGD plus BV2-Exo, as determined by CCK-8 assay. (**C**) TUNEL assay for detecting apoptosis in neurons treated with OGD or OGD plus BV2-Exo. Scale bar = 20 μm. (**D**) Lactate dehydrogenase (LDH) assay for detecting LDH activity in neurons treated with OGD or OGD plus BV2-Exo. (**E**) BV2-Exo was internalized by neurons *in vivo*, as imaged by confocal microscopy. Red color indicated exosomes (PKH26), blue color indicated nuclei (DAPI), and green color indicated neurons (MAP2). Scale bar = 25 μm. (**F**) Modified neurological severity score for mice treated with control treatment (PBS) and BV2-Exo after tMCAO. (**G**) Relative infarct volume in brains of mice treated with control treatment (PBS) and BV2-Exo after tMCAO, displayed as brain cresyl violet staining and brain tissues of ischemic mice treated with indicated treatments. Yellow dotted boxes represent the infarct areas. (**H**) Double-staining of NeuN/TUNEL in brain sections of mice treated with control treatment (PBS) and BV2-Exo after tMCAO. Red color indicated NeuN and green color indicated TUNEL staining. Scale bar = 25 μm. Data are presented as mean±SD. *, *p*<0.05. At least three replicates were available for statistical analysis in each treatment.

Then, we aimed to determine whether BV2-Exo could be internalized by neurons. BV2-Exo was cocultured with neurons, and the confocal images revealed that PKH26-labeled red BV2-Exo were found in the cytoplasm of MAP2-positive neurons labeled with green fluorescence (Figure 3A). CCK-8 and LDH assays suggested that BV2-Exo significantly increased cell viability of neurons cocultured with BV2-Exo for 24 hours, followed by OGD (Figure 3B, 3D). Apoptosis of neurons induced by OGD was also attenuated by BV2-Exo, as detected by TUNEL staining (Figure 3C). Furthermore, *in vivo* study, we found that BV2-Exo could also be taken up by neurons of the ischemic mouse brain (Figure 3E). Applying neurobehavioral testing to evaluate neurobehavioral performance in mice treated BV2-Exo, the results showed that BV2-Exo treatment attenuated behavioral deficits induced by tMCAO, compared with those treated with PBS control treatment (Figure 3F). We also observed that the mice treated with BV2-Exo after tMCAO had less infarct area and apoptosis than those in the control group (Figure 3G, 3H). Together, our observations demonstrated that BV2-Exo plays a neuroprotective role in neuronal injury induced by ischemic injury in both *in vitro* and *in vivo.*

### MiRNA-137 was associated with the neuroprotective effect of BV2-Exo

**Figure 4:**
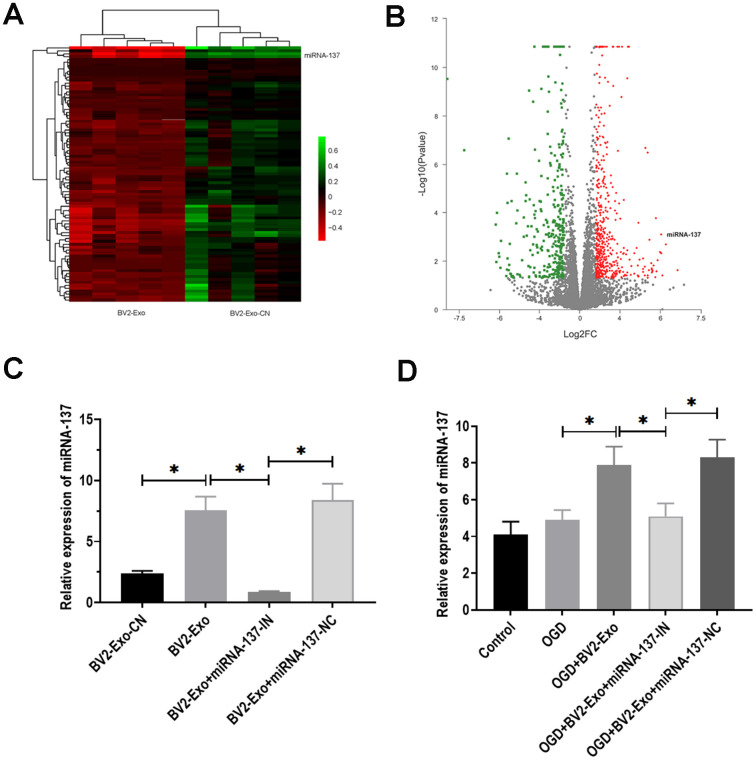
**MiRNA-137 was upregulated in M2-phenotype microglia-derived exosomes (BV2-Exo).** (**A**, **B**). Heatmap and volcano plot of expression profiles for differentially expressed miRNAs between BV2-Exo and exosomes derived from control BV2 cells (BV2 Exo-CN), as determined by RNA-seq analysis. (**C**) Expression of miRNA-137 in 1) BV2 Exo-CN, 2) BV2-Exo, 3) BV2-Exo derive from BV2 cells treated with miRNA-137 inhibitor (BV2-Exo+miRNA-137-IN), and 4) BV2-Exo derive from BV2 cells treated with miRNA-137 inhibitor negative control (BV2-Exo+miRNA-137-NC), as detected by RT-PCR. (**D**) Expression of miRNA-137 in neurons treated with 1) control, 2) OGD, 3) OGD plus BV2-Exo, 4) OGD plus BV2-Exo+miRNA-137-IN, and 5) OGD plus BV2-Exo+miRNA-137-NC, as detected by RT-PCR. Data are presented as mean±SD. *, *p*<0.05. At least three replicates were available for statistical analysis in each treatment.

Exosomal miRNAs have been demonstrated as critical functional factors in exosome-mediated intercellular communication [29]. Thus, we speculated that miRNAs might play an essential role in the neuroprotective effect of BV2-Exo. By performing RNA-seq, we identified a group of differentially expressed miRNAs between BV2-Exo and BV2 Exo-CN, as shown in heatmap and volcano plot (Figure 4A, 4B). The results revealed that miRNA-137 was one of the upregulated miRNAs. Given the essential role of miRNA-137 in IRI reported by multiple previous studies [30–32], we hypothesized that miRNA-137 might be an essential mediator for the function of BV2-Exo. Then, we applied RT-PCR assay to verify the results obtained from RNA-seq analysis, and we found that miRNA-137 was also increased in BV2-Exo, relative to BV2 Exo-CN (Figure 4C). Meanwhile, the expression of miRNA-137 was decreased in BV2-Exo derived from BV2 cells treated with miRNA-137-IN (Figure 4C). After OGD treatment, the level of miRNA-137 was increased in neurons treated with BV2-Exo, whereas decreased in those treated with BV2-Exo derived from BV2 cells treated with miRNA-137-IN (Figure 4D). Thus, these results suggested the involvement of miRNA-137 in BV2-Exo-related neuroprotection.

### BV2-Exo attenuated neuronal apoptosis induced OGD through miRNA-137

**Figure 5:**
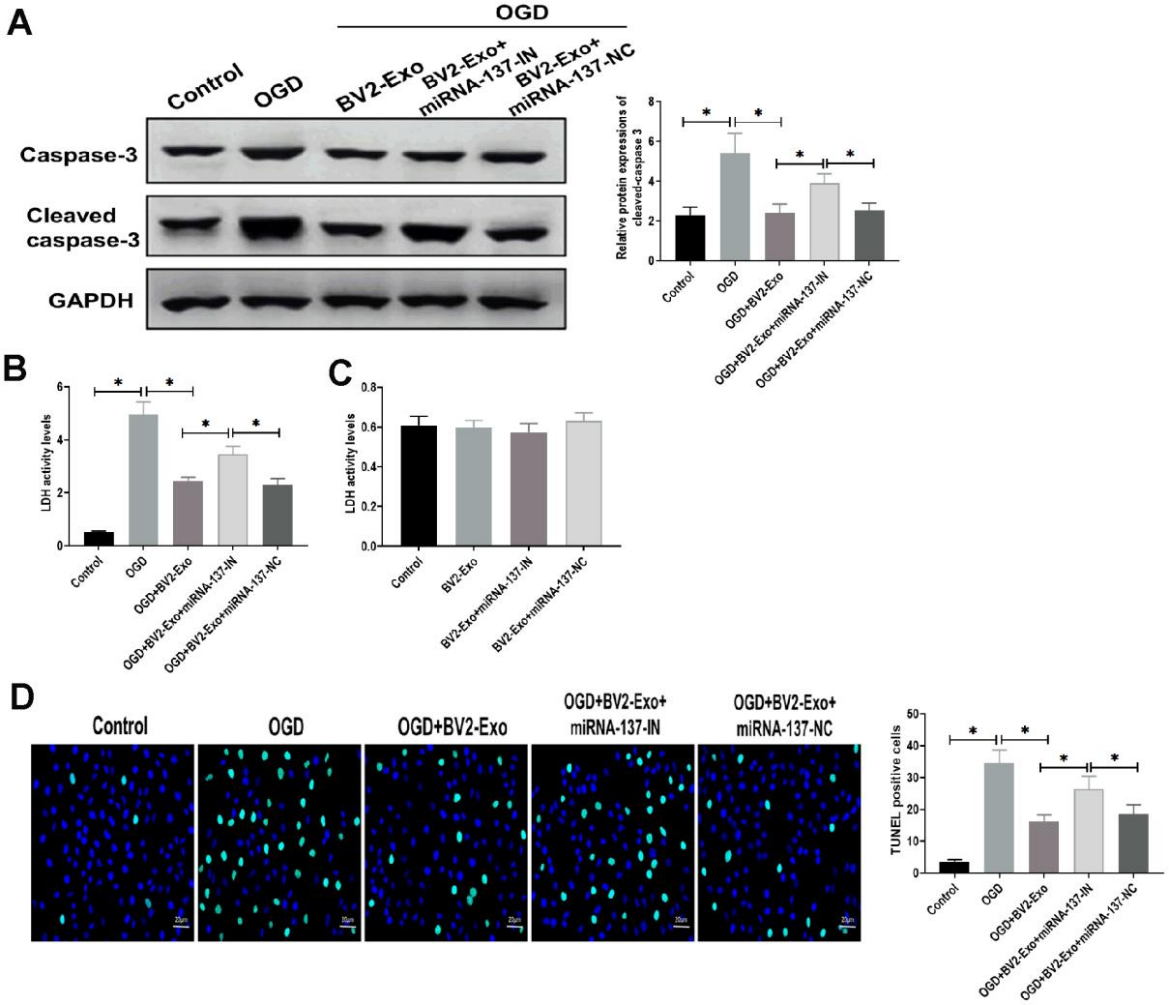
**M2-phenotype microglia-derived exosomes (BV2-Exo) attenuated neuronal apoptosis induced oxygen-glucose deprivation (OGD) through miRNA-137.** (**A**) Protein expressions of caspase-3 and cleaved caspase-3 in neurons treated with 1) control, 2) OGD, 3) OGD plus BV2-Exo, 4) OGD plus BV2-Exo+miRNA-137-IN, and 5) OGD plus BV2-Exo+miRNA-137-NC, as detected by Western blotting assay. (**B**) Lactate dehydrogenase (LDH) assay for detecting LDH activity in neurons treated with 1) control, 2) OGD, 3) OGD plus BV2-Exo, 4) OGD plus BV2-Exo+miRNA-137-IN, and 5) OGD plus BV2-Exo+miRNA-137-NC. (**C**) LDH assay for detecting LDH activity in neurons treated with 1) control, 2) BV2-Exo, 3) BV2-Exo plus BV2-Exo+miRNA-137-IN, and 4) BV2-Exo plus BV2-Exo+miRNA-137-NC. (**D**) TUNEL assay for detecting apoptosis in neurons treated with 1) control, 2) OGD, 3) OGD plus BV2-Exo, 4) OGD plus BV2-Exo+miRNA-137-IN, and 5) OGD plus BV2-Exo+miRNA-137-NC. Data are presented as mean±SD. *, *p*<0.05. At least three replicates were available for statistical analysis in each treatment.

To further determine the function of miRNA-137, we found that the expression of cleaved caspase-3 was significantly decreased in BV2-Exo-treated neurons after OGD, while partially rescued by treatment of BV2-Exo derived from BV2 cells treated with miRNA-137-IN (Figure 5A). For LDH assay, LDH activity was increased by OGD treatment, which was reversed by BV2-Exo and BV2-Exo derived from BV2 cells treated with miRNA-137-IN with different degrees (Figure 5B). Notably, we did not observe the different effects of these treatments on LDH activity in neurons under normal conditions (Figure 5C). Furthermore, TUNEL assay revealed that BV2-Exo with reduced expression of miRNA-137 could also partially reverse the effect of BV2-Exo on apoptosis after OGD (Figure 5D). Together, these results suggested that miRNA-137 participates in, at least in part, the neuroprotective effect of BV2-Exo.

### MiRNA-137 mediated the function of BV2-Exo through targeting Notch1

**Figure 6:**
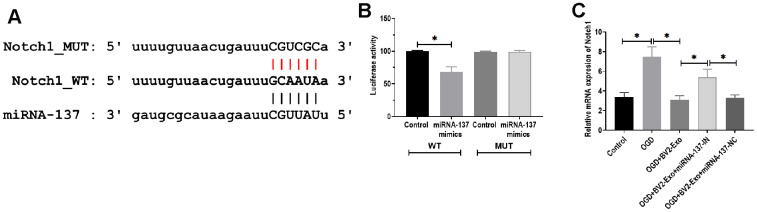
**MiRNA-137 directly targeted Notch1.** (**A**) Putative binding site in 3’UTR of Notch1, as predicted by bioinformatics software. (**B**) Luciferase reporter assay of miRNA-137 targeting on 3’UTR of Notch1. (**C**) mRNA expression of Notch1 in neurons treated with 1) control, 2) OGD, 3) OGD plus BV2-Exo, 4) OGD plus BV2-Exo+miRNA-137-IN, and 5) OGD plus BV2-Exo+miRNA-137-NC, as detected by RT-PCR. Data are presented as mean±SD. *, *p*<0.05. At least three replicates were available for statistical analysis in each treatment.

**Figure 7:**
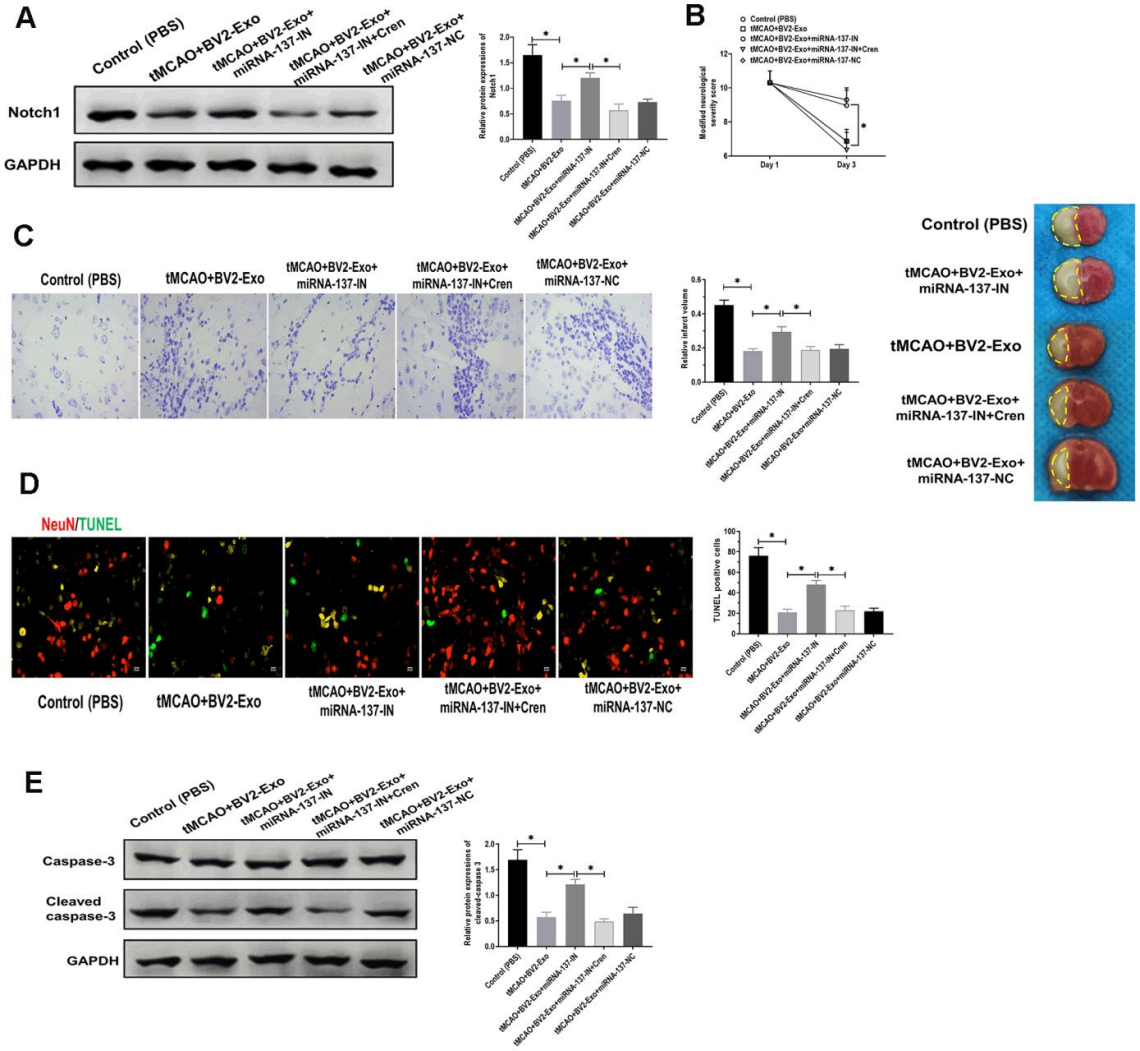
**M2-phenotype microglia-derived exosomes (BV2-Exo) attenuated tMCAO-induced neuronal apoptosis through Notch1.** (**A**) Protein expression of Notch1 in brains of mice treated with 1) control, 2) tMCAO plus BV2-Exo, 3) tMCAO plus BV2-Exo+miRNA-137-IN, 4) tMCAO plus BV2-Exo+miRNA-137-IN and Crenigacestat, and 5) tMCAO plus BV2-Exo+miRNA-137-NC, as detected by Western blotting assay. (**B**) Modified neurological severity score for mice treated with 1) control, 2) tMCAO plus BV2-Exo, 3) tMCAO plus BV2-Exo+miRNA-137-IN, 4) tMCAO plus BV2-Exo+miRNA-137-IN and Crenigacestat, and 5) tMCAO plus BV2-Exo+miRNA-137-NC. (**C**) Relative infarct volume in brains of mice treated with 1) control, 2) tMCAO plus BV2-Exo, 3) tMCAO plus BV2-Exo+miRNA-137-IN, 4) tMCAO plus BV2-Exo+miRNA-137-IN and Crenigacestat, and 5) tMCAO plus BV2-Exo+miRNA-137-NC, displayed as brain cresyl violet staining and brain tissues of ischemic mice treated with indicated treatments. Yellow dotted boxes represent the infarct areas. (**D**) Double-staining of NeuN/TUNEL in brain sections of mice treated with 1) control, 2) tMCAO plus BV2-Exo, 3) tMCAO plus BV2-Exo+miRNA-137-IN, 4) tMCAO plus BV2-Exo+miRNA-137-IN and Crenigacestat, and 5) tMCAO plus BV2-Exo+miRNA-137-NC. Red color indicated NeuN and green color indicated TUNEL staining. Scale bar = 25 μm. (**E**) Protein expression of caspase-3 and cleaved caspase-3 in brains of mice treated with 1) control, 2) tMCAO plus BV2-Exo, 3) tMCAO plus BV2-Exo+miRNA-137-IN, 4) tMCAO plus BV2-Exo+miRNA-137-IN and Crenigacestat, and 5) tMCAO plus BV2-Exo+miRNA-137-NC, as detected by Western blotting assay. Data are presented as mean±SD. *, *p*<0.05. At least three replicates were available for statistical analysis in each treatment.

To determine the downstream targeting gene of miRNA-137, we performed bioinformatics analysis in several databases, including MiRanda (MicroRNA.org)) and TargetScan (http://www.targetscan.org). The results showed that there was a potential binding site of miRNA-137 in 3’UTR of Notch1 (Figure 6A). Through performing luciferase reporter assay, the results showed that the luciferase activity was decreased in 293T cells co-transfected with miRNA-137 mimics and luciferase reporter plasmids containing wild-type miRNA-137 binding site, compared with those treated with plasmids containing mutant miRNA-137 binding site (Figure 6B). Also, we found that BV2-Exo significantly decreased mRNA expression of Notch1, which was partially reversed by inhibition of miRNA-137 expression in BV2-Exo (Figure 6C). To further investigate the function of Notch1 in the ischemic mouse model, BV2-Exo, BV2-Exo with reduced expression of miRNA-137, and BV2-Exo with reduced expression of miRNA-137 plus Cren (a selective inhibitor of Notch1) were administrated to ischemic mice, respectively. The results demonstrated that protein expression of Notch1 was decreased in the brains of mice treated with BV2-Exo and BV2-Exo with reduced expression of miRNA-137 plus Cren, while increased in mice treated with BV2-Exo with reduced expression of miRNA-137 (Figure 7A). Functionally, decreased expression of Notch1 induced by BV2-Exo was associated with decreased neurobehavioral deficits, fewer infarct areas in the brain, and less apoptosis (Figure 7B–7D). In contrast, the elevation of Notch1 induced by the downregulation of miRNA-137 led to more severe behavioral deficits, more infarct areas, and more apoptosis (Figure 7B–7D). We also noticed that the effect of increased Notch1 was reversed by Notch1 inhibitor Cren. Furthermore, the expression of cleaved caspase-3 was decreased in mice treated with BV2-Exo and BV2-Exo with reduced expression of miRNA-137 plus Cren while increased in mice treated with BV2-Exo with reduced expression of miRNA-137, compared with those in the control group (Figure 7E). Collectively, these findings suggested that neuroprotective role of BV2-Exo was mediated through miRNA-137/Notch1 pathway.

## DISCUSSION

Microglial colonize the CNS and play essential roles in brain development and maintaining the function of CNS [33], primarily including 1) the housekeeping function for maintaining neuronal health and normal physiological function; 2) the sentinel function for the constant perception of environmental changes; as well as 3) defensive function required for neuroprotection [2, 3]. It has been demonstrated that microglial dominate sites of CNS injury in which they promote both injury and repair [34]. Distinct microglial subsets may cause these different effects, i.e., “classically activated” pro-inflammatory (M1) or “alternatively activated” anti-inflammatory (M2) cells [7, 35]. Accumulating evidence shows that microglia in M2 phenotype display notable neuroprotective effect in many brain diseases, such as cerebral ischemia [24], intracerebral hemorrhage [36], and Parkinson's disease [25]. As such, investigating the mechanism underlying the neuroprotective effect of M2-phenotype microglia would make a significant contribution to the treatment of brain injuries and diseases. In this study, we reported that exosomes secreted by microglia BV2 cells in M2 phenotype were internalized by neurons that were subjected to ischemic injury, thereby promoting the survival of ischemic neurons through exosomal miRNA-137 via Notch1 pathway.

Over the last decade, numerous studies demonstrate that the effect of exosomes in intercellular communication constitutes an essential mechanism for local and systematic intercellular transportation of bioactive cargos, such as proteins, DNA, mRNAs, long noncoding RNAs (lncRNAs), and miRNAs, which can significantly regulate the physiological processes in the recipient cells [37, 38]. Given these critical roles, exosome-mediated therapeutic strategies have received growing attention and applied in many diseases studies, including cancers [39], cardiovascular diseases [40, 41], and metabolic disease [42]. Meanwhile, microglia-derived exosomes also display essential roles in the development and progression of CNS diseases [43]. For example, Chang et al. reported that alpha-synuclein-induced exosomes express higher levels of major histocompatibility complex (MHC) class II molecules and membrane tumor necrosis factor alpha (TNF-alpha) and promote neuron apoptosis, suggesting that microglial exosomes might be critical mediators for neurodegeneration in Parkinson's disease [44]. A recent study reported by Li et al. suggested that TBI-induced increase of miRNA-124-3p in microglial exosomes suppresses neuronal autophagy, thereby proecting injured nerve [45]. In addition, exosomes generated from lipopolysaccharide (LPS)-activated microglia promote neuron apoptosis, which can reversed by the knockdown of TNF-inducible protein 3 (TNFAIP3) [45]. In the present study, we observed that M2-phenotype BV2 cell-conditioned medium could reduce neuronal death and apoptosis following OGD, in which BV2-Exo is an essential factor mediating the neuroprotective role of BV2 cell-conditioned medium. Together, microglial exosomes exert critical role in the neuroprotection in the CNS diseases, including ischemic injury.

It has been well documented that miRNAs are tightly involved in the regulation of diverse physiological and pathological processes [46, 47]. For ischemic brain injury, the crucial neuroprotective roles of miRNAs have uncovered in many previous studies. For instance, miRNA-21 and miRNA29b are unregulated in neurons, whereas miRNA-30b, miRNA-137, and miRNA-107 are downregulated in astrocytes in the presence of OGC treatment, suggesting the distinct role of each miRNA in different types of neural cells under ischemic condition [48]. Zhao et al. demonstrated that miRNA-23a-3p attenuates ischemic injury in mice treated with focal cerebral ischemia-reperfusion, which associated with an upregulated level of superoxide dismutase and reduced production of reactive nitrogen species [49]. Furthermore, Stary et al. reported that the inhibition of miRNA-200c leads to a significant reduction in infarct volume and neurological deficit through targeting reelin, indicating miRNA-200c is a potential biological target for mitigating injury induced by transient cerebral ischemia [50]. In the present study, RNA-seq and RT-PCR analysis revealed that miRNA-137 was upregulated in BV2-Exo, relative to BV2-Exo-CN. Furthermore, the knockdown of miRNA-137 could partially reverse the neuroprotective effect of BV2-Exo. Collectively, these results indicate the involvement of miRNA-137 in the role of microglial exosomes. MiRNA-137 is abundantly expressed in the CNS and participates in the regulation of neuron maturation and differentiation [30, 31]. Li et al. demonstrated that miRNA-137 is a hypoxia-responsive miRNA to inhibit mitophagy through targeting mitophagy receptors NIX and FUNDC1 [32]. In addition, miRNA-137 exerts an inhibitory effect on anesthesia-induced neurodegeneration and memory loss in the hippocampus through silencing CDC42 [51]. Taken together, these results indicate that miRNA-137 is an essential miRNA to modulate protective responses in the brain.

To further investigate the mechanism in which microglia exosomal miRNA-317 plays a neuroprotective role in response to ischemic injury, we applied bioinformatics analysis and luciferase reporter assay to determine the downstream targeting gene of exosomal miRNA-317. Our results revealed that Notch1 was a direct target of miRNA-137 and that BV2-Exo derived from miRNA-137-knockdown BV2 cells plus Notch1 inhibitor Crenigacestat exerts a similar neuroprotective role as BV2-Exo alone. Our observations are consistent with a previous study reporting that miRNA-137 protects neurons from OGD-induced injury via targeting Notch1 signaling pathway [52]. It has been demonstrated that Notch signaling pathway plays a critical role in ischemia-associated injury in various physiological systems, such as the kidney [53] and heart [54]. In the CNS, the upregulation of miRNA-210 is associated with angiogenesis in ischemic brain cortexes of adult rats through the involvement of Notch1 signaling [55]. LncRNA GAS5 promotes mouse ischemic stroke through acting as a competing endogenous RNA (ceRNA) for miRNA-137 to regulate Notch1 signaling [56]. Also, Li et al. reported the importance of miRNA-137/Notch1 pathway in hypoxia-associated retinal injury [57]. Notably, to the best of our best knowledge, we first reported that Notch1 acts as a downstream target of exosomal miRNA in ischemic injured neurons, suggesting that Notch signaling is also essential for intercellular communication between injured cells and the surrounding microenvironment. These observations might also provide a potential target for developing treatments for ischemic injury in the brain.

With regard to the limits of this study, the first should be addressed is the cell line used in our study. Although BV2 cells are an immortalized neonatal mouse microglia cells, which is a universal surrogate of primary microglia *in vitro* experiments [58], BV2 cells have a different gene expression profile than primary microglia and differentially behave in response to stimuli [59]. Thus, the results obtained from BV2 cells can not be applied entirely in primary microglia. Secondly, due to the relatively small sample size *in vivo* experiments, the results of these experiments should be interpreted with caution until more samples are used to study further.

In conclusion, the results suggest that microglial exosomes could alleviate ischemic brain injury *in vitro* and *in vivo* through transporting exosomal miRNA-137 and its targeting gene Notch1. This study provides novel insight into the development of exosomal miRNAs-based therapeutic strategies for ischemic brain injury.

## MATERIALS AND METHODS

### Cell culture

BV2 cells were purchased from Elabscience (Wuhan, China) and cultured in Dulbecco's modified Eagle medium (DMEM) (Sigma-Aldrich, Shanghai, China) supplemented with 10% Fetal Bovine Serum (FBS) (Thermo Fisher Scientific, Waltham, USA), 1% streptomycin, and 1% penicillin (Thermo Fisher Scientific, Waltham, USA). BV2 cells were subjected to interleukin 4 (IL-4) (20 ng/mL) for 48 hours to convert to M2 phenotype, which was verified using PCR and Western blotting assay to detect M2 microglia markers. After identification, BV2 M2 cells were seeded in culture dishes for 24 hours, and the medium was collected and centrifuged at 500×g for 5 min. The supernatant was collected as the conditioned medium (BV2-CM) for subsequent experiments. Primary cortical neurons were collected from C57BL/6 mice at 16-18 embryonic days (Department of Laboratory Animal Science, Fudan University, Shanghai) as previously described [60]. Primary neurons were cultured in an incubator at 37° C in 5% CO_2,_ and the medium was replaced every three days. Primary neurons were used for subsequent experiments between 7 and 10 days in culture.

### Oxygen-glucose deprivation (OGD) treatment

Before OGD treatment, primary cortical neurons were treated with 20 μg exosomes (BV2-Exo) or BV2-Exo-CN for 24 hours. Oxygen-glucose deprivation (OGD) treatments were performed in a sealed chamber filled with 95% N_2_ and 5% CO_2_. During OGD treatment, cells were cultured in deoxygenated glucose-free DMEM (Thermo Fisher Scientific, Waltham, USA). After 45-min OGD treatment, cells were moved to a regular incubator to recover for 12 hours, and the medium was replaced with normal maintenance medium.

### Real-time PCR

Total RNA was isolated from exosomes or brain tissues using PolyATtract® mRNA Isolation Systems (Promega, Madison, USA) according to the manufacturer's instruction. RNA concentration and purity were determined using NanoDrop 2000 (Thermo Fisher Scientific, Waltham, USA) according to the manufacturer's instruction. Reverse transcription was performed using GoScript™ Reverse Transcription System (Promega, Madison, USA) according to the manufacturer's instruction. RT-PCR assay was performed using GoTaq® Green Master Mix (Promega, Madison, USA) in 7900 HT PCR system (ABI, Foster City, USA) according to the manufacturer's instructions. The primer information was list in Table 1. The expressions of GAPDH and U6 were used as control. The relative expressions were calculated using 2^-ΔΔCt^ method [61].

**Table 1:** Primer information.

**Genes**	**Primers sequences**
CD206	F: 5’- AAGGCGGTGACCTCACAAG -3’
CD206	R: 5’- AAAGTCCAATTCCTCGATGGTG -3’
ARG	F: 5’- CAAGCTGGGAATTGGCAAAG -3’
ARG	R: 5’- GGTCCAGTCCATCAACATCAA -3’
Notch1	F: 5’- GCAGTTGTGCTCCTGAAGAA -3’
Notch1	R: 5’- CGGGCGGCCAGAAAC -3’
GAPDH	F: 5’- AAGGTGAAGGTCGGAGTCAAC -3’
GAPDH	R: 5’- GGGGTCATTGATGGCAACAATA -3’
U6	F: 5'- CTCGCTTCGGCAGCACA -3'
U6	R: 5’- AACGCTTCACGAATTTGCGT -3’
MiRNA-137	5’- CTACAAAGGGAAGCCCTTTC -3’

### Western blotting

Total protein was isolated from the ipsilateral hemisphere samples, and the amount of protein was measured using Pierce™ BCA Protein Assay Kit (Thermo Fisher Scientific, Waltham, USA). The procedure of Western blotting was performed as previously described [62]. The primary antibodies were as follows: CD63 (1:1000), Tsg101 (1:1000), CD206 (1:1000) (Abcam, Shanghai, China); caspase-3 (1:1000), cleaved caspase-3 (1:1000), ARG (1:200), Notch1 (1:500), and GAPDH (1:1000) (BD Biosciences, San Jose, USA). Proteins were determined using enhanced chemiluminescence (MilliporeSigma, Burlington, USA) and photographed using a Molecular Imager VersaDoc 4000 system (Bio-Rad, Hercules, USA).

### Cell transfection, luciferase reporter assay and exosome labeling

BV2 cells (2 × 10^6^ cells) were transfected with lentiviruses containing miRNA-137 inhibitor (miRNA-137-IN) (OriGene, Rockville, USA) for 72 hours, according to the manufacturer's instructions. Lentivirus containing negative control shRNA was used as a control (miRNA-137-NC). HEK 293T cells (2 × 10^6^ cells) (Sigma-Aldrich, Shanghai, China) were co-transfected with miRNA-137 mimics (RiboBio, Guangzhou, China) and luciferase reporter plasmids containing wild-type or a mutant miRNA-137 binding site in 3’UTR of Notch1 using the Lipofectamine™ 3000 Transfection Reagent (Invitrogen, Waltham, MA, USA) according to the manufacturer’s instructions. At 24 hours post-transfection, relative luciferase activities were measured using the dual-luciferase reporter assay kit (Promega, Madison, USA). Exosomes were fluorescently labeled using the PKH26 Fluorescent Cell Linker Kits according to the manufacturer's instructions (Sigma-Aldrich, Shanghai, China).

### Exosome isolation and identification

Exosomes (BV2-Exo) were isolated from the culture supernatant of M2 BV2 cells using the ultracentrifugation method, as previously described [63]. Briefly, donor cell M2 BV2 cells were washed with PBS solution twice, and the medium was replaced with exosome-free medium (ultracentrifugation at 100,000 g for 16 hours) for 48 hours. Then, the supernatant was collected and was subjected to serial ultracentrifugation at 2000×g for 30 min, 10000×g for 30 min, and 100000×g for 60 min at 4° C. Next, exosomes were washed with PBS solution once at 100000×g for 60 min and were suspended for further identification. Exosomes (BV2-Exo-CN) isolated from BV2 without being treated IL-4 were used as control. The protein content of exosomes was measured using Pierce™ BCA Protein Assay Kit (Thermo Fisher Scientific, Waltham, USA). Exosomal markers CD63 and TSG101 were determined by Western blotting assay. Then, transmission electron microscope (TEM) (Philips, Amsterdam, Netherlands) and nanoparticle tracking analysis were performed to verify the identification of exosomes, as previously described [63].

### RNA-seq

Total RNA and cDNA of BV2-Exo and BV2-Exo-CN were prepared as described above. The procedure of RNA-seq was performed on Illumina Hiseq2500 platform according to the manufacturer's instructions Majorbio Biotech Co., Ltd. (Shanghai, China). RNA-seq analysis was performed as previously described [64] and the instruction provided by Majorbio Biotech Co., Ltd. The clean reads were obtained through quality control and then blasted against the Rfam database to annotate the miscellaneous RNAs [65]. After filtering snRNA, scRNA, rRNA, and other non-coding RNA, the remaining sRNAs were aligned to the miRBase version 21.0 to identify the known and novel miRNAs. Then, the differential miRNA expression profile was calculated by the package DESeq2 [66] and edgeR [67]. MiRNAs with |log2^fold change^| ≥2 and *P*_adj_-value <0.05 were identified as statistical differences. Volcano plots and heatmaps of differential miRNA expression profiles were plotted by R Package TRAPR [68].

### Immunohistochemistry and TUNEL assay

Immunohistochemistry assay was performed as previously described [69, 70]. The primary antibodies used were as follows: CD206 (1:500), AGR (1:500), MAP2 (1:500) (Abcam, Shanghai, China). The double-staining of TUNEL/DAPI was performed for *in vitro* study using TUNEL Assay Kit according to the manufacturer's instructions (Abcam, Shanghai, China). The double-staining of NeuN/TUNEL was performed for the infarct area of brain sections for *in vivo* study (Abcam, Shanghai, China). For the quantification of apoptotic cells, the stained cells or sections were viewed under a fluorescence microscope (Olympus, Tokyo, Japan). The mean of TUNEL-positive cells at least 5 random microscopic regions were counted for each sample by two independent lab technicians.

### CCK-8 and lactate dehydrogenase (LDH) assay

Cell viability was determined using Cell Counting Kit-8 (CCK-8) according to the manufacturer's instructions (Dojindo, Kumamoto, Japan). Absorbance was determined at 450 nm using a microplate reader (BioTek, Winooski, USA). Lactate dehydrogenase (LDH) assay was performed to determine the death rate of primary neurons using LDH assay kit according to the manufacturer's instructions (Abcam, Shanghai, China).

### *In vivo* ischemic mouse model

All experiments performed on animals were approved by the Institutional Animal Care and Use Committee of Heilongjiang University of Traditional Chinese Medicine. Thirty-six C57BL/6 male mice (6-8 weeks old; 24-28 g) (Department of Laboratory Animal Science, Fudan University, Shanghai) were randomly divided into six groups (n=12). Except for mice in the sham group (n=12), the other thirty mice were subjected to transient middle cerebral artery occlusion (tMCAO) surgery, as previously described [71]. To verify the success of surgery, the interruption and reperfusion of surface cerebral blood flow was confirmed using transcranial laser Doppler (DRK4, Devon, UK). After surgery, mice were randomly divided into five groups (n=12), including 1) PBS group: mice were injected with PBS (200 μl); 2) BV2-Exo group: mice were injected with BV2-Exo; 3) BV2-Exo-miRNA-137-IN group: mice were injected with exosomes derived from BV2 cells treated with miRNA-137-IN; 4) BV2-Exo-miRNA-137-IN+ Crenigacestat group: mice were co-injected with exosomes derived from BV2 cells treated with miRNA-137-IN and 100 μg/kg Crenigacestat (Cren) (LY3039478) [72]. 5) miRNA-137-NC group: mice were injected with exosomes derived from BV2 cells treated with miRNA-137-NC. The injection of exosomes (100 μg/day) was immediately performed through the tail vein after the surgery and lasted for three consecutive days. Three days after surgery, six mice in each group were used for detecting RNA and protein expressions, and the other six mice were used for immunohistochemistry assay. Infarct volume was evaluated as previously described [73]. Briefly, the brain was sectioned in a cryostat into a series of 20 μm sections and strained with 0.1% cresyl violet. The ratio of staining in the ipsilateral and contralateral hemispheres was determined using ImageJ [74]. The influence of postischemic edema to the infarct volume was corrected for swelling by comparing nonischemic and ischemic hemispheres, as previously described [75, 76]. The infarct volume was calculated through the formula:

V=∑h/3[ΔSn+(ΔSn∗ΔSn+1)1/2+ΔSn+1],

where *V* indicates volume, *h* indicates the distance between the two adjacent sections, contralateral area subtracts normal area of the ipsilateral hemisphere was calculated as the infarct area ΔS, andΔ*;Sn* and Δ*Sn+1* indicates the different area between the two adjacent sections [77].

### Neurobehavioral testing

To determine neurobehavioral performance, all mice underwent modified neurological severity score (mNSS) testing 1 day before and 3 days after tMCAO surgery, in which motor, reflex, and balance functions were evaluated. The evaluation score was from a normal score 0 to the most severe deficit score 14 [78].

### Statistical analysis

Data were represented as mean ± standard deviation (SD). Comparisons between groups were performed using ANOVA and Student's t-test. Statistical analysis was performed using SPSS 13.0 (SPSS, Inc., Chicago, USA). *p*<0.05 was regarded as statistical difference.

## References

[1] El Khoury J. Neurodegeneration and the neuroimmune system. Nat Med. 2010; 16:1369–70. 10.1038/nm1210-1369 21135838

[2] Salter MW, Stevens B. Microglia emerge as central players in brain disease. Nat Med. 2017; 23:1018–27. 10.1038/nm.4397 28886007

[3] Hickman S, Izzy S, Sen P, Morsett L, El Khoury J. Microglia in neurodegeneration. Nat Neurosci. 2018; 21:1359–69. 10.1038/s41593-018-0242-x 30258234 PMC6817969

[4] Wolf SA, Boddeke HW, Kettenmann H. Microglia in physiology and disease. Annu Rev Physiol. 2017; 79:619–43. 10.1146/annurev-physiol-022516-034406 27959620

[5] Stepanova A, Kahl A, Konrad C, Ten V, Starkov AS, Galkin A. Reverse electron transfer results in a loss of flavin from mitochondrial complex I: potential mechanism for brain ischemia reperfusion injury. J Cereb Blood Flow Metab. 2017; 37:3649–58. 10.1177/0271678X17730242 28914132 PMC5718331

[6] Hu X, Li P, Guo Y, Wang H, Leak RK, Chen S, Gao Y, Chen J. Microglia/macrophage polarization dynamics reveal novel mechanism of injury expansion after focal cerebral ischemia. Stroke. 2012; 43:3063–70. 10.1161/STROKEAHA.112.659656 22933588

[7] Orihuela R, McPherson CA, Harry GJ. Microglial M1/M2 polarization and metabolic states. Br J Pharmacol. 2016; 173:649–65. 10.1111/bph.13139 25800044 PMC4742299

[8] Cacci E, Ajmone-Cat MA, Anelli T, Biagioni S, Minghetti L. *In vitro* neuronal and glial differentiation from embryonic or adult neural precursor cells are differently affected by chronic or acute activation of microglia. Glia. 2008; 56:412–25. 10.1002/glia.20616 18186084

[9] Walton NM, Sutter BM, Laywell ED, Levkoff LH, Kearns SM, Marshall GP 2nd, Scheffler B, Steindler DA. Microglia instruct subventricular zone neurogenesis. Glia. 2006; 54:815–25. 10.1002/glia.20419 16977605

[10] Kigerl KA, Gensel JC, Ankeny DP, Alexander JK, Donnelly DJ, Popovich PG. Identification of two distinct macrophage subsets with divergent effects causing either neurotoxicity or regeneration in the injured mouse spinal cord. J Neurosci. 2009; 29:13435–44. 10.1523/JNEUROSCI.3257-09.2009 19864556 PMC2788152

[11] Théry C, Ostrowski M, Segura E. Membrane vesicles as conveyors of immune responses. Nat Rev Immunol. 2009; 9:581–93. 10.1038/nri2567 19498381

[12] Sato-Kuwabara Y, Melo SA, Soares FA, Calin GA. The fusion of two worlds: non-coding RNAs and extracellular vesicles—diagnostic and therapeutic implications (review). Int J Oncol. 2015; 46:17–27. 10.3892/ijo.2014.2712 25338714 PMC4238728

[13] Hu G, Drescher KM, Chen XM. Exosomal miRNAs: biological properties and therapeutic potential. Front Genet. 2012; 3:56. 10.3389/fgene.2012.00056 22529849 PMC3330238

[14] Huang S, Ge X, Yu J, Han Z, Yin Z, Li Y, Chen F, Wang H, Zhang J, Lei P. Increased miR-124-3p in microglial exosomes following traumatic brain injury inhibits neuronal inflammation and contributes to neurite outgrowth via their transfer into neurons. FASEB J. 2018; 32:512–28. 10.1096/fj.201700673R 28935818

[15] Ge X, Guo M, Hu T, Li W, Huang S, Yin Z, Li Y, Chen F, Zhu L, Kang C, Jiang R, Lei P, Zhang J. Increased microglial exosomal miR-124-3p alleviates neurodegeneration and improves cognitive outcome after rmTBI. Mol Ther. 2020; 28:503–22. 10.1016/j.ymthe.2019.11.017 31843449 PMC7001001

[16] Xu W, Wu Y, Hu Z, Sun L, Dou G, Zhang Z, Wang H, Guo C, Wang Y. Exosomes from microglia attenuate photoreceptor injury and neovascularization in an animal model of retinopathy of prematurity. Mol Ther Nucleic Acids. 2019; 16:778–90. 10.1016/j.omtn.2019.04.029 31163320 PMC6545376

[17] Thind A, Wilson C. Exosomal miRNAs as cancer biomarkers and therapeutic targets. J Extracell Vesicles. 2016; 5:31292. 10.3402/jev.v5.31292 27440105 PMC4954869

[18] Bartel DP. MicroRNAs: genomics, biogenesis, mechanism, and function. Cell. 2004; 116:281–97. 10.1016/s0092-8674(04)00045-5 14744438

[19] Paul P, Chakraborty A, Sarkar D, Langthasa M, Rahman M, Bari M, Singha RS, Malakar AK, Chakraborty S. Interplay between miRNAs and human diseases. J Cell Physiol. 2018; 233:2007–18. 10.1002/jcp.25854 28181241

[20] Croce CM, Calin GA. miRNAs, cancer, and stem cell division. Cell. 2005; 122:6–7. 10.1016/j.cell.2005.06.036 16009126

[21] Viñas JL, Burger D, Zimpelmann J, Haneef R, Knoll W, Campbell P, Gutsol A, Carter A, Allan DS, Burns KD. Transfer of microRNA-486-5p from human endothelial colony forming cell-derived exosomes reduces ischemic kidney injury. Kidney Int. 2016; 90:1238–50. 10.1016/j.kint.2016.07.015 27650731

[22] Xiao J, Pan Y, Li XH, Yang XY, Feng YL, Tan HH, Jiang L, Feng J, Yu XY. Cardiac progenitor cell-derived exosomes prevent cardiomyocytes apoptosis through exosomal miR-21 by targeting PDCD4. Cell Death Dis. 2016; 7:e2277. 10.1038/cddis.2016.181 27336721 PMC5143405

[23] Zhao L, Jiang X, Shi J, Gao S, Zhu Y, Gu T, Shi E. Exosomes derived from bone marrow mesenchymal stem cells overexpressing microRNA-25 protect spinal cords against transient ischemia. J Thorac Cardiovasc Surg. 2019; 157:508–17. 10.1016/j.jtcvs.2018.07.095 30224076

[24] Liu X, Wen S, Yan F, Liu K, Liu L, Wang L, Zhao S, Ji X. Salidroside provides neuroprotection by modulating microglial polarization after cerebral ischemia. J Neuroinflammation. 2018; 15:39. 10.1186/s12974-018-1081-0 29426336 PMC5807735

[25] Subramaniam SR, Federoff HJ. Targeting microglial activation states as a therapeutic avenue in Parkinson’s disease. Front Aging Neurosci. 2017; 9:176. 10.3389/fnagi.2017.00176 28642697 PMC5463358

[26] Francos-Quijorna I, Amo-Aparicio J, Martinez-Muriana A, López-Vales R. IL-4 drives microglia and macrophages toward a phenotype conducive for tissue repair and functional recovery after spinal cord injury. Glia. 2016; 64:2079–92. 10.1002/glia.23041 27470986

[27] Liu C, Li Y, Yu J, Feng L, Hou S, Liu Y, Guo M, Xie Y, Meng J, Zhang H, Xiao B, Ma C. Targeting the shift from M1 to M2 macrophages in experimental autoimmune encephalomyelitis mice treated with fasudil. PLoS One. 2013; 8:e54841. 10.1371/journal.pone.0054841 23418431 PMC3572131

[28] Essandoh K, Yang L, Wang X, Huang W, Qin D, Hao J, Wang Y, Zingarelli B, Peng T, Fan GC. Blockade of exosome generation with GW4869 dampens the sepsis-induced inflammation and cardiac dysfunction. Biochim Biophys Acta. 2015; 1852:2362–71. 10.1016/j.bbadis.2015.08.010 26300484 PMC4581992

[29] Umezu T, Ohyashiki K, Kuroda M, Ohyashiki JH. Leukemia cell to endothelial cell communication via exosomal miRNAs. Oncogene. 2013; 32:2747–55. 10.1038/onc.2012.295 22797057

[30] Chen L, Wang X, Wang H, Li Y, Yan W, Han L, Zhang K, Zhang J, Wang Y, Feng Y, Pu P, Jiang T, Kang C, Jiang C. miR-137 is frequently down-regulated in glioblastoma and is a negative regulator of cox-2. Eur J Cancer. 2012; 48:3104–11. 10.1016/j.ejca.2012.02.007 22406049

[31] Sun G, Ye P, Murai K, Lang MF, Li S, Zhang H, Li W, Fu C, Yin J, Wang A, Ma X, Shi Y. miR-137 forms a regulatory loop with nuclear receptor TLX and LSD1 in neural stem cells. Nat Commun. 2011; 2:529. 10.1038/ncomms1532 22068596 PMC3298567

[32] Li W, Zhang X, Zhuang H, Chen HG, Chen Y, Tian W, Wu W, Li Y, Wang S, Zhang L, Chen Y, Li L, Zhao B, et al. MicroRNA-137 is a novel hypoxia-responsive microRNA that inhibits mitophagy via regulation of two mitophagy receptors FUNDC1 and NIX. J Biol Chem. 2014; 289:10691–701. 10.1074/jbc.M113.537050 24573672 PMC4036186

[33] Chen Z, Trapp BD. Microglia and neuroprotection. J Neurochem. 2016 (Suppl 1); 136:10–17. 10.1111/jnc.13062 25693054

[34] Jin X, Yamashita T. Microglia in central nervous system repair after injury. J Biochem. 2016; 159:491–96. 10.1093/jb/mvw009 26861995

[35] Tang Y, Le W. Differential roles of M1 and M2 microglia in neurodegenerative diseases. Mol Neurobiol. 2016; 53:1181–94. 10.1007/s12035-014-9070-5 25598354

[36] Lin L, Yihao T, Zhou F, Yin N, Qiang T, Haowen Z, Qianwei C, Jun T, Yuan Z, Gang Z, Hua F, Yunfeng Y, Zhi C. Inflammatory regulation by driving microglial M2 polarization: neuroprotective effects of cannabinoid receptor-2 activation in intracerebral hemorrhage. Front Immunol. 2017; 8:112. 10.3389/fimmu.2017.00112 28261199 PMC5306140

[37] Schorey JS, Cheng Y, Singh PP, Smith VL. Exosomes and other extracellular vesicles in host-pathogen interactions. EMBO Rep. 2015; 16:24–43. 10.15252/embr.201439363 25488940 PMC4304727

[38] Meldolesi J. Exosomes and ectosomes in intercellular communication. Curr Biol. 2018; 28:R435–44. 10.1016/j.cub.2018.01.059 29689228

[39] Hannafon BN, Ding WQ. Intercellular communication by exosome-derived microRNAs in cancer. Int J Mol Sci. 2013; 14:14240–69. 10.3390/ijms140714240 23839094 PMC3742242

[40] Cervio E, Barile L, Moccetti T, Vassalli G. Exosomes for intramyocardial intercellular communication. Stem Cells Int. 2015; 2015:482171. 10.1155/2015/482171 26089917 PMC4454760

[41] Emanueli C, Shearn AI, Angelini GD, Sahoo S. Exosomes and exosomal miRNAs in cardiovascular protection and repair. Vascul Pharmacol. 2015; 71:24–30. 10.1016/j.vph.2015.02.008 25869502 PMC4838026

[42] Huang-Doran I, Zhang CY, Vidal-Puig A. Extracellular vesicles: novel mediators of cell communication in metabolic disease. Trends Endocrinol Metab. 2017; 28:3–18. 10.1016/j.tem.2016.10.003 27810172

[43] Paolicelli RC, Bergamini G, Rajendran L. Cell-to-cell communication by extracellular vesicles: focus on microglia. Neuroscience. 2019; 405:148–57. 10.1016/j.neuroscience.2018.04.003 29660443

[44] Chang C, Lang H, Geng N, Wang J, Li N, Wang X. Exosomes of BV-2 cells induced by alpha-synuclein: important mediator of neurodegeneration in PD. Neurosci Lett. 2013; 548:190–95. 10.1016/j.neulet.2013.06.009 23792198

[45] Li D, Huang S, Yin Z, Zhu J, Ge X, Han Z, Tan J, Zhang S, Zhao J, Chen F, Wang H, Lei P. Increases in miR-124-3p in microglial exosomes confer neuroprotective effects by targeting FIP200-mediated neuronal autophagy following traumatic brain injury. Neurochem Res. 2019; 44:1903–23. 10.1007/s11064-019-02825-1 31190315

[46] Cai Y, Yu X, Hu S, Yu J. A brief review on the mechanisms of miRNA regulation. Genomics Proteomics Bioinformatics. 2009; 7:147–54. 10.1016/S1672-0229(08)60044-3 20172487 PMC5054406

[47] Huang Y, Shen XJ, Zou Q, Wang SP, Tang SM, Zhang GZ. Biological functions of microRNAs: a review. J Physiol Biochem. 2011; 67:129–39. 10.1007/s13105-010-0050-6 20981514

[48] Ziu M, Fletcher L, Rana S, Jimenez DF, Digicaylioglu M. Temporal differences in microRNA expression patterns in astrocytes and neurons after ischemic injury. PLoS One. 2011; 6:e14724. 10.1371/journal.pone.0014724 21373187 PMC3044134

[49] Zhao H, Tao Z, Wang R, Liu P, Yan F, Li J, Zhang C, Ji X, Luo Y. MicroRNA-23a-3p attenuates oxidative stress injury in a mouse model of focal cerebral ischemia-reperfusion. Brain Res. 2014; 1592:65–72. 10.1016/j.brainres.2014.09.055 25280466

[50] Stary CM, Xu L, Sun X, Ouyang YB, White RE, Leong J, Li J, Xiong X, Giffard RG. MicroRNA-200c contributes to injury from transient focal cerebral ischemia by targeting reelin. Stroke. 2015; 46:551–56. 10.1161/STROKEAHA.114.007041 25604249 PMC4346276

[51] Huang C, Zhang X, Zheng J, Chen C, Chen Y, Yi J. Upregulation of miR-137 protects anesthesia-induced hippocampal neurodegeneration. Int J Clin Exp Pathol. 2014; 7:5000–07. 25197371 PMC4152061

[52] Shi F, Dong Z, Li H, Liu X, Liu H, Dong R. MicroRNA-137 protects neurons against ischemia/reperfusion injury through regulation of the notch signaling pathway. Exp Cell Res. 2017; 352:1–8. 10.1016/j.yexcr.2017.01.015 28132879

[53] Kobayashi T, Terada Y, Kuwana H, Tanaka H, Okado T, Kuwahara M, Tohda S, Sakano S, Sasaki S. Expression and function of the Delta-1/Notch-2/Hes-1 pathway during experimental acute kidney injury. Kidney Int. 2008; 73:1240–50. 10.1038/ki.2008.74 18418349

[54] Zhou XL, Wan L, Xu QR, Zhao Y, Liu JC. Notch signaling activation contributes to cardioprotection provided by ischemic preconditioning and postconditioning. J Transl Med. 2013; 11:251. 10.1186/1479-5876-11-251 24098939 PMC3853230

[55] Lou YL, Guo F, Liu F, Gao FL, Zhang PQ, Niu X, Guo SC, Yin JH, Wang Y, Deng ZF. miR-210 activates notch signaling pathway in angiogenesis induced by cerebral ischemia. Mol Cell Biochem. 2012; 370:45–51. 10.1007/s11010-012-1396-6 22833359

[56] Chen F, Zhang L, Wang E, Zhang C, Li X. LncRNA GAS5 regulates ischemic stroke as a competing endogenous RNA for miR-137 to regulate the Notch1 signaling pathway. Biochem Biophys Res Commun. 2018; 496:184–90. 10.1016/j.bbrc.2018.01.022 29307821

[57] Li H, Zhu Z, Liu J, Wang J, Qu C. MicroRNA-137 regulates hypoxia-induced retinal ganglion cell apoptosis through Notch1. Int J Mol Med. 2018; 41:1774–82. 10.3892/ijmm.2017.3319 29286063

[58] Stansley B, Post J, Hensley K. A comparative review of cell culture systems for the study of microglial biology in Alzheimer’s disease. J Neuroinflammation. 2012; 9:115. 10.1186/1742-2094-9-115 22651808 PMC3407712

[59] Timmerman R, Burm SM, Bajramovic JJ. An overview of *in vitro* methods to study microglia. Front Cell Neurosci. 2018; 12:242. 10.3389/fncel.2018.00242 30127723 PMC6087748

[60] Rubiolo J, Vale C, Martín V, Méndez A, Boente-Juncal A. Transcriptomic Profiling of Mice Primary Cortical Neurons in Response to Medium Change. Transcriptomics. 2016; 4:2. 10.4172/2329-8936.1000138

[61] Schmittgen TD, Livak KJ. Analyzing real-time PCR data by the comparative C(T) method. Nat Protoc. 2008; 3:1101–08. 10.1038/nprot.2008.73 18546601

[62] Lang HL, Hu GW, Chen Y, Liu Y, Tu W, Lu YM, Wu L, Xu GH. Glioma cells promote angiogenesis through the release of exosomes containing long non-coding RNA POU3F3. Eur Rev Med Pharmacol Sci. 2017; 21:959–72. 28338200

[63] Wu L, Zhang X, Zhang B, Shi H, Yuan X, Sun Y, Pan Z, Qian H, Xu W. Exosomes derived from gastric cancer cells activate NF-κB pathway in macrophages to promote cancer progression. Tumour Biol. 2016; 37:12169–80. 10.1007/s13277-016-5071-5 27220495

[64] Sun Y, Luo G, Zhao L, Huang L, Qin Y, Su Y, Yan Q. Integration of RNAi and RNA-seq reveals the immune responses of Epinephelus coioides to sigX gene of Pseudomonas plecoglossicida. Front Immunol. 2018; 9:1624. 10.3389/fimmu.2018.01624 30061893 PMC6054955

[65] Duchesne R, Bouffartigues E, Oxaran V, Maillot O, Bénard M, Feuilloley MG, Orange N, Chevalier S. A proteomic approach of SigX function in pseudomonas aeruginosa outer membrane composition. J Proteomics. 2013; 94:451–59. 10.1016/j.jprot.2013.10.022 24332064

[66] Love MI, Huber W, Anders S. Moderated estimation of fold change and dispersion for RNA-seq data with DESeq2. Genome Biol. 2014; 15:550. 10.1186/s13059-014-0550-8 25516281 PMC4302049

[67] Robinson MD, McCarthy DJ, Smyth GK. edgeR: a bioconductor package for differential expression analysis of digital gene expression data. Bioinformatics. 2010; 26:139–40. 10.1093/bioinformatics/btp616 19910308 PMC2796818

[68] Lim JH, Lee SY, Kim JH. TRAPR: R package for statistical analysis and visualization of RNA-seq data. Genomics Inform. 2017; 15:51–53. 10.5808/GI.2017.15.1.51 28416950 PMC5389949

[69] Wang J, Matias J, Gilbert ER, Tachibana T, Cline MA. Hypothalamic mechanisms associated with corticotropin-releasing factor-induced anorexia in chicks. Neuropeptides. 2019; 74:95–102. 10.1016/j.npep.2019.01.003 30739813

[70] Xin H, Katakowski M, Wang F, Qian JY, Liu XS, Ali MM, Buller B, Zhang ZG, Chopp M. MicroRNA cluster miR-17-92 Cluster in Exosomes Enhance Neuroplasticity and Functional Recovery After Stroke in Rats. Stroke. 2017; 48:747–753. 10.1161/strokeaha.116.015204 28232590 PMC5330787

[71] Yang G, Chan PH, Chen J, Carlson E, Chen SF, Weinstein P, Epstein CJ, Kamii H. Human copper-zinc superoxide dismutase transgenic mice are highly resistant to reperfusion injury after focal cerebral ischemia. Stroke. 1994; 25:165–70. 10.1161/01.str.25.1.165 8266365

[72] Mancarella S, Serino G, Dituri F, Cigliano A, Ribback S, Wang J, Chen X, Calvisi DF, Giannelli G. Crenigacestat, a selective NOTCH1 inhibitor, reduces intrahepatic cholangiocarcinoma progression by blocking VEGFA/DLL4/MMP13 axis. Cell Death Differ. 2020; 27:2330–43. 10.1038/s41418-020-0505-4 32042099 PMC7370218

[73] Park EM, Cho S, Frys KA, Glickstein SB, Zhou P, Anrather J, Ross ME, Iadecola C. Inducible nitric oxide synthase contributes to gender differences in ischemic brain injury. J Cereb Blood Flow Metab. 2006; 26:392–401. 10.1038/sj.jcbfm.9600194 16049426

[74] Abràmoff MD, Magalhães PJ, Ram SJ. Image processing with ImageJ. Biophotonics International. 2004; 11:36–42.

[75] Cho S, Park EM, Febbraio M, Anrather J, Park L, Racchumi G, Silverstein RL, Iadecola C. The class B scavenger receptor CD36 mediates free radical production and tissue injury in cerebral ischemia. J Neurosci. 2005; 25:2504–12. 10.1523/JNEUROSCI.0035-05.2005 15758158 PMC6725161

[76] Park EM, Cho S, Frys K, Racchumi G, Zhou P, Anrather J, Iadecola C. Interaction between inducible nitric oxide synthase and poly(ADP-ribose) polymerase in focal ischemic brain injury. Stroke. 2004; 35:2896–901. 10.1161/01.STR.0000147042.53659.6c 15514191

[77] Shan HM, Zang M, Zhang Q, Shi RB, Shi XJ, Mamtilahun M, Liu C, Luo LL, Tian X, Zhang Z, Yang GY, Tang Y, Pu J, Wang Y. Farnesoid X receptor knockout protects brain against ischemic injury through reducing neuronal apoptosis in mice. J Neuroinflammation. 2020; 17:164. 10.1186/s12974-020-01838-w 32450881 PMC7249620

[78] Li Y, Chopp M, Chen J, Wang L, Gautam SC, Xu YX, Zhang Z. Intrastriatal transplantation of bone marrow nonhematopoietic cells improves functional recovery after stroke in adult mice. J Cereb Blood Flow Metab. 2000; 20:1311–19. 10.1097/00004647-200009000-00006 10994853

